# FUS/TLS Is a Co-Activator of Androgen Receptor in Prostate Cancer Cells

**DOI:** 10.1371/journal.pone.0024197

**Published:** 2011-09-01

**Authors:** Simon Haile, Aaron Lal, Jae-Kyung Myung, Marianne D. Sadar

**Affiliations:** Genome Sciences Centre, BC Cancer Agency, Vancouver, British Columbia, Canada; University of Kentucky College of Medicine, United States of America

## Abstract

Androgen receptor (AR) is a member of the nuclear receptor family of transcription factors. Upon binding to androgens, AR becomes transcriptionally active to regulate the expression of target genes that harbor androgen response elements (AREs) in their promoters and/or enhancers. AR is essential for the growth and survival of prostate cancer cells and is therefore a target for current and next-generation therapeutic modalities against prostate cancer. Pathophysiologically relevant protein-protein interaction networks involving AR are, however, poorly understood. In this study, we identified the protein FUsed/Translocated in LipoSarcoma (FUS/TLS) as an AR-interacting protein by co-immunoprecipitation of endogenous proteins in LNCaP human prostate cancer cells. The hormonal response of FUS expression in LNCaP cells was shown to resemble that of other AR co-activators. FUS displayed a strong intrinsic transactivation capacity in prostate cancer cells when tethered to basal promoters using the GAL4 system. Chromatin immunoprecipitation experiments showed that FUS was recruited to ARE III of the enhancer region of the *PSA* gene. Data from ectopic overexpression and “knock-down” approaches demonstrated that AR transcriptional activity was enhanced by FUS. Depletion of FUS reduced androgen-dependent proliferation of LNCaP cells. Thus, FUS is a novel co-activator of AR in prostate cancer cells.

## Introduction

Androgen receptor (AR) is required for the survival and growth of prostate cancer cells. Accordingly, advanced prostate cancer can be treated with androgen ablation therapies. Unfortunately, these therapies eventually fail and the disease becomes lethal, castration-resistant prostate cancer (CRPC). The most widely supported mechanism underlying CRPC involves AR. The amino-terminus domain (NTD) of AR is required for both ligand-dependent and ligand-independent activity of the receptor (for a review see [1]). Thus, current efforts to develop more effective drugs against CRPC are focused on more effective blockage of androgen synthesis, antiandrogens that competitively bind the AR ligand-binding domain (LBD) with much higher affinity [2], and inhibitors of the AR NTD [3]. Continued efforts to develop drugs would benefit from improved understanding of the protein-protein interaction networks involving the AR. To this end, we employed a proteomic approach and identified novel endogenous AR interaction partners in prostate cancer cells that include members of a group of proteins called FET/TET. This family of proteins includes: FUsed in Sarcoma (FUS)/Translocated in LipoSarcoma (TLS), EWig's Sarcoma protein (EWS), and the TATA binding Protein-Associated Factor, TAF15.

FET proteins display diverse functions including transcriptional modulation, splicing, cell spreading, and DNA repair. FET proteins have similar transcriptional activation domains (TADs) at their NTD and RNA recognition motif (RRM) and repeats of the tripeptide RGG at their carboxyl terminus [4], [5]. The TAD of FET proteins contains XYXXQ-rich motif, X being a small amino acid (Gly, Ala, Ser or Pro) [6]. This motif is also shared with the proto-oncoprotein SYT, the human nuclear receptor co-activator SYT-interacting Protein/Co-activator Activator (SIP/CoAA), and SWI/SNF/BAF250 [6]. FET NTDs, including the potent TADs, are fused to DNA-binding domains (DBDs) of various transcription factors in a subclass of sarcomas and other cancers [5], which leads to hyperactivation of corresponding transcriptional activity. Emerging evidence suggests that native, full-length FET proteins bind to and activate the functions of specific transcription factors in part by recruiting co-factors such as CBP/p300 [4]–[5].

## Results

### AR and FUS are found in the same protein complex

**Figure 1:**
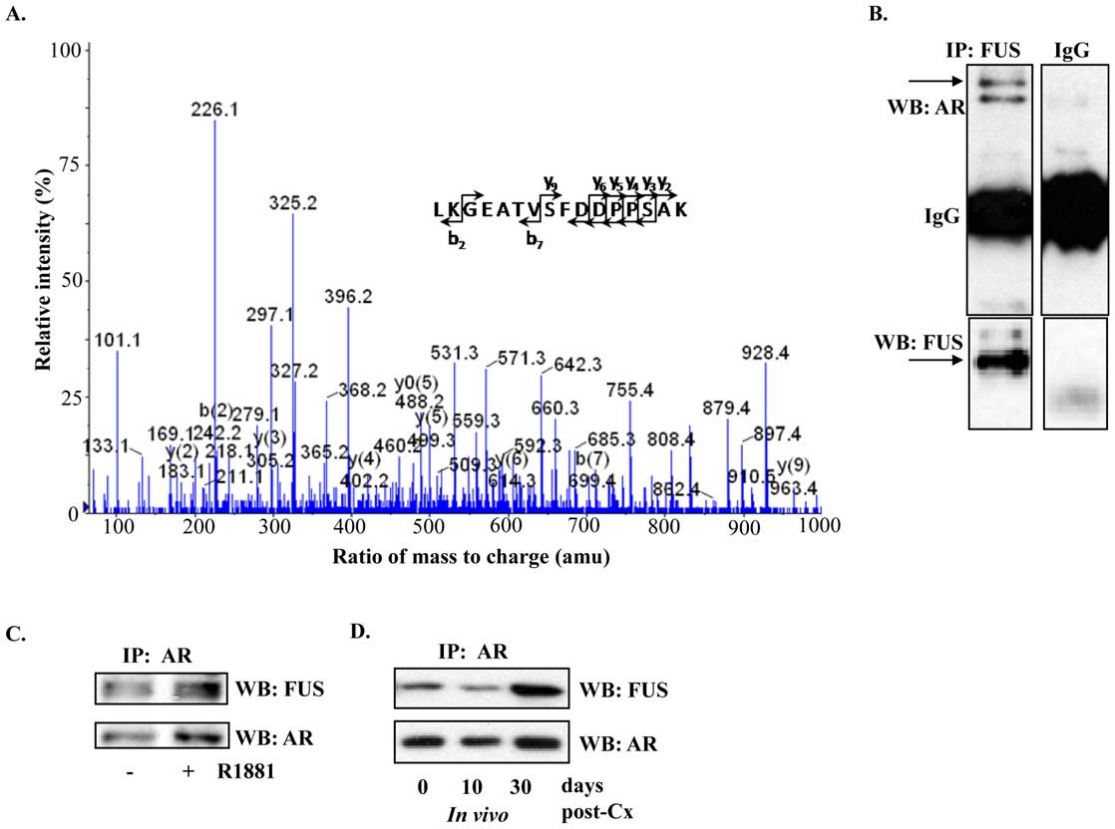
AR and FUS are found in the same complex in LNCaP cells. (**A**) Identification of FUS as an AR-interacting protein by co-immunopreciptation (Co-IP) followed by mass spectrometry (MS). MS/MS spectra of m/z 1660.77 (from 831.39, 2+) of FUS and two others shown in supplementary material were unambiguously assigned the identified sequence LKGEATVSFDDPPSAK, APKPDGPGGGPGGSHMGGNYGDDR, and TGQPMINLYTDR, respectively. (**B**) Validation of AR-FUS interaction using Co-IP followed by western blot analysis. LNCaP cells in culture were induced with 10 nM R1881 for 6 hrs and whole lysates were used for IP with anti-FUS antibody followed by anti-AR western blot (WB) analysis. (**C**) Reverse IP with anti-AR followed by WB with anti-FUS antibody. (**D**) IP of endogenous complexes of AR and FUS from subcutaneous LNCaP xenografts. Tumours were harvested from intact mice (I), 10 days after castration (C10) or 30 days after castration (C30). Lysates from these tumors were individually used for IP with anti-AR antibody followed by WB with indicated antibodies.

LNCaP human prostate cancer cell line [7] recapitulates important features of prostate cancer including expression of both prostate-specific antigen (PSA) and AR as well as sensitivity to androgen. For our initial screen, we used nuclear lysates from LNCaP cells that were treated with the synthetic androgen R1881 (10 nM) for 3 hrs. The lysates were immunoprecipitated with anti-AR antibody and samples were subjected to multidimensional protein identification technology (MudPIT) [8]. Mass spectrometric analysis identified FUS, which belongs to the FET family of proteins interacting with AR (Figure 1 A, Figure S1, Figure S2 and Table 1). Co-immunoprecipitation (co-IP) experiments followed by Western blot analysis validated interaction between AR and FUS (Figure 1B). AR co-immunoprecipitated (co-IP) with FUS, whereas immunoprecipitation with isotype IgG control did not pull-down AR or FUS. Reverse co-IP with anti-AR followed by Western blot analysis with anti-FUS antibody also showed that AR interacted with FUS (Figure 1C). Consistent with previous reports, treatment with R1881 enhanced levels of AR protein. *In vivo* association of FUS with AR was confirmed using extracts of LNCaP xenografts that were grown in mice before or after castration (Figure 1D). Together these data support that AR and FUS associate within the same complex in prostate cancer cells.

**Table 1:** Analysis of mass spectrometric data for FUS and AR.

Acc^a^	Desc^b^	Ex_mz^c^	Ex_z^d^	Ex_mr^e^	Score^f^	Miss^g^	Sequence^h^
IPI00221354	FUS	704.8295	2	1407.6445	57.44	0	TGQPMINLYTDR
		751.6276	3	2251.8608	63.47	0	APKPDGPGGGPGGSHM- GGNYGDDR
		831.3902	2	1660.7658	40.59	1	LKGEATVSFDDPPSAK
IPI00333533	AR	574.2814	2	1146.5482	40.82	0	VPYPSPTCVK
		669.8026	2	1337.5906	72.36	0	SGALDEAAAYQSR
		778.3500	2	1554.6855	102.57	0	DNYLGGTSTISDNAK
		1076.9564	2	2151.9054	110.15	0	LQEEGEASSTTSPTEET- TQK

a Acc, protein accession number searched in IPI_human database;

b Desc, protein description;

c Ex_mz, experimental peptides m/z value;

d Exp_z, experimental peptides charge state;

e Ex_mr, experimental peptides molecular weight;

f Score, peptide score acquired from Mascot search;

g Miss, the number of missing cleavage;

h Sequence, Identified peptide sequences.

### Subcellular localization of FUS in prostate cancer cells

**Figure 2:**
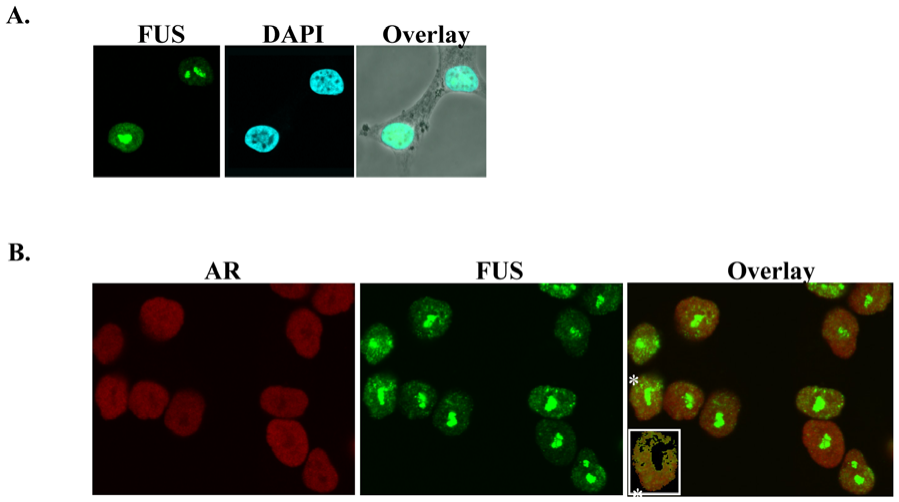
Subcellular localization of FUS in prostate cancer cells. (**A**) Nuclear localization of FUS in LNCaP cells. Cells were treated with R1881 (10 nM) for 3 hrs. Confocal immunofluoresence was performed using anti-FUS antibody (green) and cells were counterstained with DAPI (blue). (**B**) Colocalization of AR and FUS. Dual staining shows co-localization of AR (red) with FUS (green) in LNCaP cells treated with 10 nM R1881 for 3 hrs. The inset in the right panel represents the cell designated with (*) after threshold gating to remove the bright central green spot.

FET proteins can be localized divergently in the nucleus and/or cytoplasm [5], [9]. Immunofluorescence microscopy using antibodies targeted against endogenous proteins was performed in LNCaP cells. FUS was exclusively localized to the nucleus (Figure 2A). FUS appeared to be localized in distinct speckles within the nucleus (Figure 2A, B). Heterogeneity in sub-nuclear distribution of FUS amongst the cell population was observed (Figure 2A, B). Dual staining showed co-localization of AR with FUS (Figure 2B) in LNCaP cells that were treated with 10 nM R1881 for 3 hrs. Pearson's coefficient approached 0.8 in some cells within specific regions of nuclei.

### FUS has intrinsic transactivation activity in prostate cancer cells

**Figure 3:**
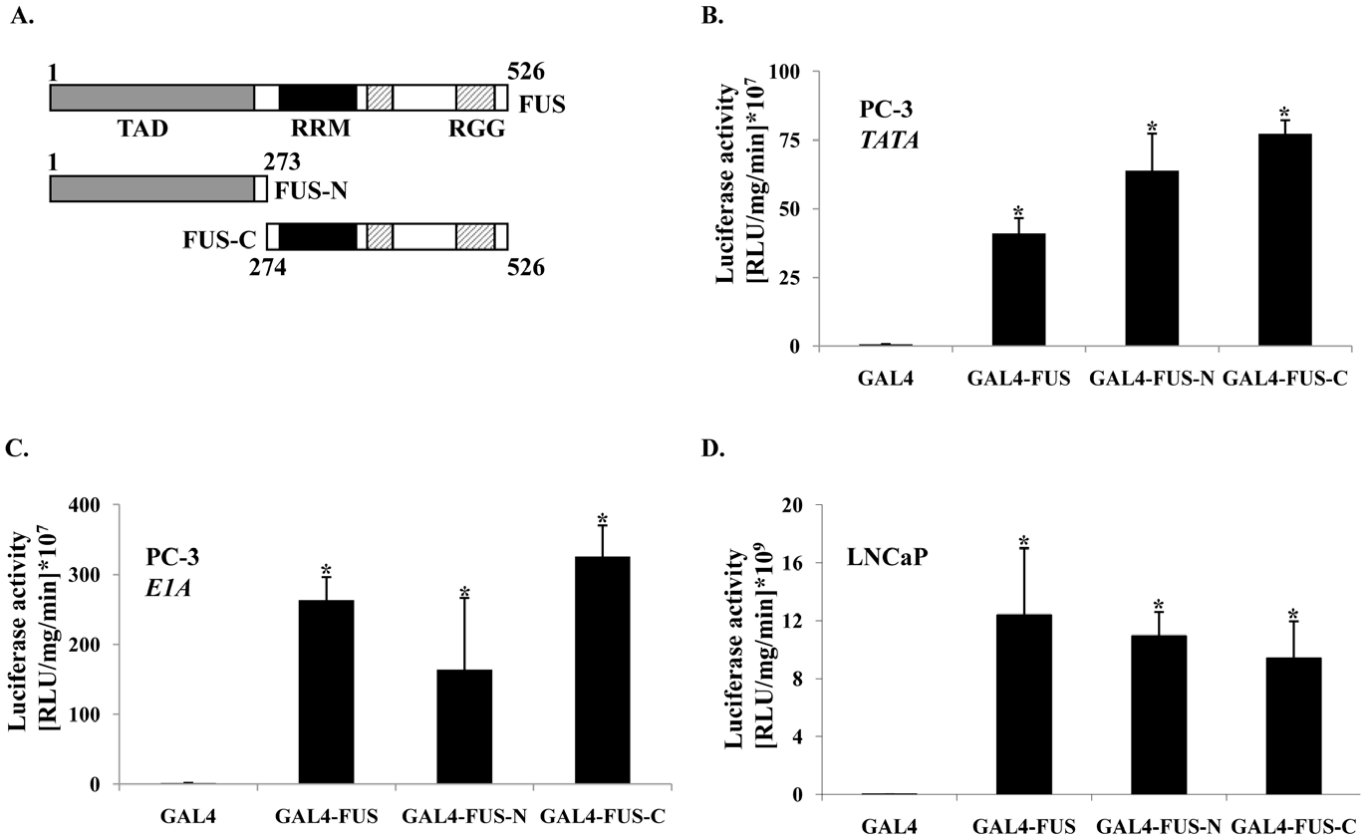
Intrinsic transactivation potential of FUS in prostate cancer cells. (**A**) Chimeric constructs of full-length FUS, N- or C-fragments of FUS fused to the Gal4 DBD. TAD = Transactivation domain; RRM = RNA recognition motif; RGG = repeats of a tripeptide containing arginine and two glycines. (**B**) Transactivation activity of FUS in PC3 prostate cancer cells. PC-3 cells in 6-well plates were co-transfected with 50–100 ng of Gal4 chimera, and 1 ug of the reporter gene pFR-LUC that contains GAL4 binding sites and a TATA element. GAL4 DBD alone served as a negative control. 2 ug PGL2 empty plasmid was added to all DNA mixes. (**C**) As in (B) but the reporter plasmid contained Gal4-binding sites and TATA element juxtaposed into E1a promoter. (**D**) As in (B) but with LNCaP instead of PC-3 cells. Columns = mean ± standard deviation. *P<0.05, n = 3.

The TADs of FET proteins can confer robust transcriptional transactivation in some tumors [4]–[5], but this has not been explored in prostate cancer cells. Here, we employed the GAL4 transactivation assay to explore if transactivation activity could be attributed to FUS in prostate cancer cells. Chimeric constructs harboring full-length FUS (or fragments therein) fused to the Gal4-DBD were used for this assay (Figure 3A). PC3 prostate cancer cells, devoid of functional AR, were co-transfected with each of the Gal4 fusion constructs, and a reporter gene containing the minimal Gal4-binding sites (p5×Gal4UAS-TATA-luciferase). GAL4-DBD alone was used as a negative control and it had minimal activity (Figure 3B). In contrast, Gal4-FUS induced luciferase activity significantly (Figure 3B) which was consistent with it having intrinsic transactivation activity. To begin to delineate which domain (s) account for this intrinsic transactivation capacity, we employed two constructs. The first construct had the NTD transactivation domain (FUS-N) and the other contained the carboxyl terminal RRM and RGGs (FUS-C) (Figure 3A). Both fragments displayed activities that were comparable to full-length FUS (Figure 3B). To assess the promoter context dependency of these activities, we employed a different reporter containing the minimal GAL4 binding sites and a TATA element, which were juxtaposed into the E1a promoter. Similar, albeit generally higher, transactivation capacity was observed in the context of this promoter as in the minimal promoter (Figure 3C). Strong transactivation capacity of the GAL4-FUS fusions was also demonstrated in LNCaP cells (Figure 3D).

### FUS promotes AR transcriptional activity in prostate cancer cells

**Figure 4:**
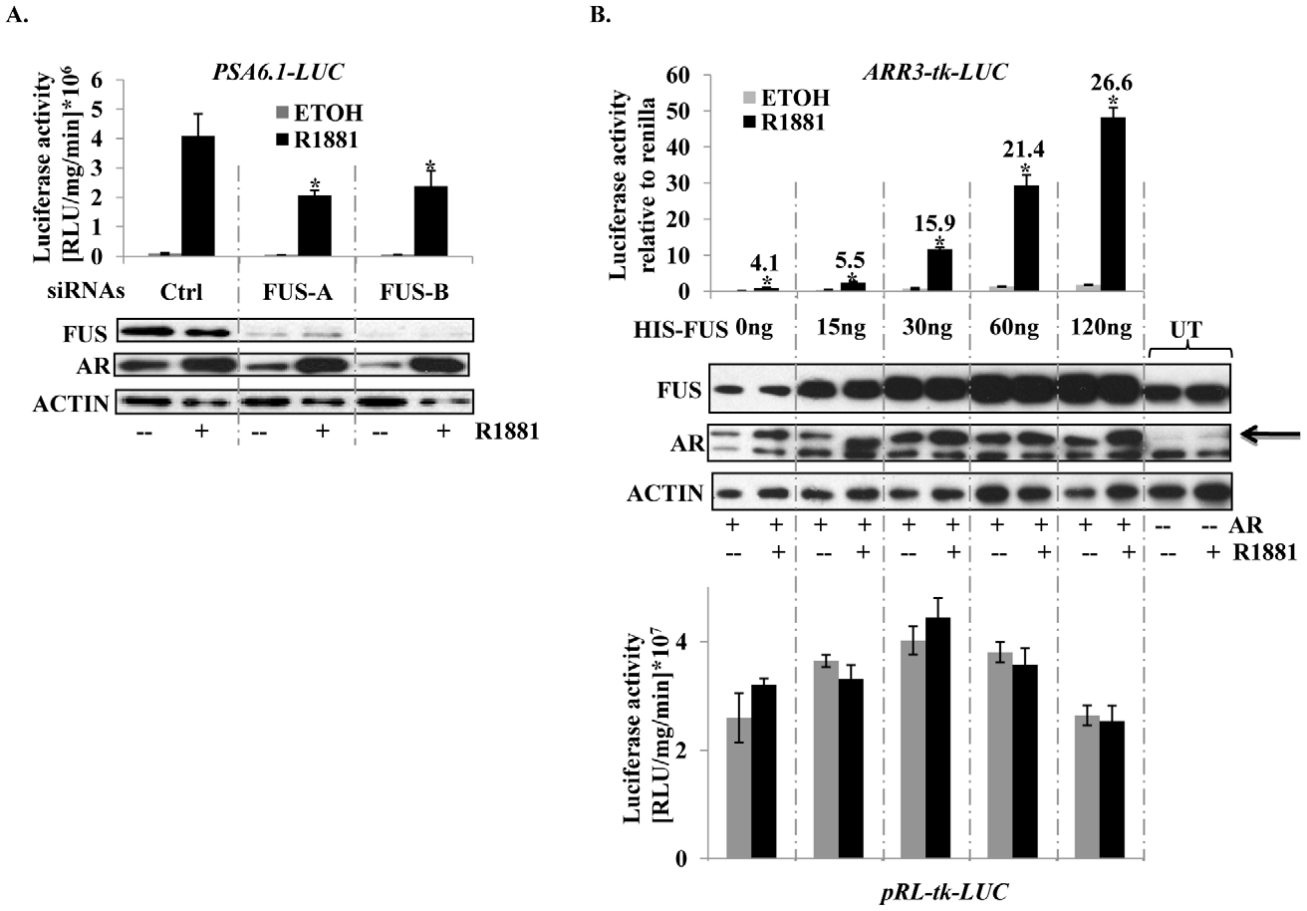
Levels of FUS alter AR transcriptional activity. (**A**) Depletion of FUS correlates with decreased AR activity. Levels of FUS were decreased in LNCaP cells using siRNAs targeting two different regions of the respective mRNAs and subsequently transfected with 1 ug of plasmid for PSA(6.1)-luciferase. 2 ug p-LUC empty plasmid was added to all siRNA/DNA mixes. Amounts are per well in a 6-well plate. Cells were treated with 10 nM R1881 for 24 hrs. Western blot analysis of the corresponding samples show levels of FUS, AR, and Actin. (**B**) Ectopic expression of FUS enhances AR activity. 293FT cells in a 48 well plate were transfected with 15 ng AR expression plasmid, 620 ng ARR3-tk-luciferase reporter plasmid, 62 ng renilla luciferase plasmid and indicated amounts of His-FUS expression plasmid. Luciferase activities were normalized with those from renilla luciferase that contains the minimal TK promoter. Values over each black bar represent fold-induction by R1881 after normalization (upper panel). Western blot analysis of the corresponding samples show levels of FUS, AR, and ACTIN (middle panel). UT: untransfected cells. Renilla luciferase activity from the same samples normalized to total protein served as a specificity control (lower panel). Columns = mean ± standard deviation. *P<0.05, n = 3.

Having demonstrated a transactivation capacity of FUS that was independent of the promoter in prostate cancer cells as described above, we next evaluated the involvement of FUS specifically in AR transcriptional activity at target genes. Two different siRNAs targeting independent regions of the cognate mRNA were employed to deplete the levels of FUS. AR activity was measured in co-transfected LNCaP cells using the PSA (6.1 kb)-luciferase reporter gene construct. This reporter contains the PSA promoter and enhancer regions harboring several AREs that confer induction by androgens [10]–[11] in an AR-dependent manner [12]–[13]. Relative to the control, R1881 induction of luciferase activity was significantly reduced upon depletion of FUS without reducing levels of AR (Figure 4A). To corroborate these findings with an over-expression approach, we co-transfected AR expression vector, the ARR3-tk-LUC reporter plasmid, and various amounts of a plasmid allowing His-tagged FUS expression into HEK293 cells. The ARR3-tk-LUC reporter contains six AREs and is highly inducible by androgens. Overexpression of FUS increased AR transcriptional activity measured as luciferase activity from ARR3-tk-LUC in a dose-dependent manner (Figure 4B, upper and middle panels). His-FUS did not significantly alter the transcriptional activity at the pRL-tk-LUC, which solely contains the thymidine kinase (tk) basal promoter (Figure 4B, lower panel).

**Figure 5:**
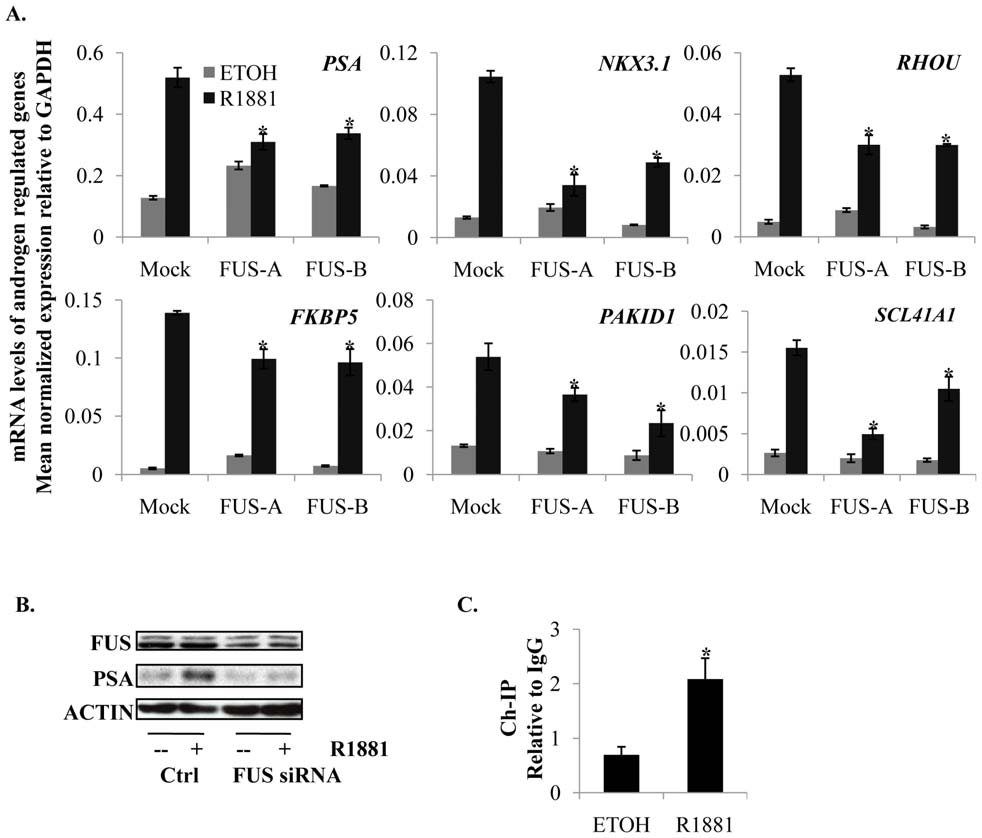
FUS enhances endogenous expression of AR target genes. (**A**) mRNA levels of androgen regulated genes. siRNA mediated depletion of FUS decreases cognate mRNA for PSA and several other AR target genes. qRT-PCR was used to measure mRNA levels. Values represent ratios of GAPDH-normalized signals to those derived from mock-transfected LNCaP cells. Cells were treated for 24 hrs following siRNA transfection. (**B**) PSA protein levels. LNCaP cells were treated for 8 hrs with 10 nM R1881 or vehicle following siRNA transfection and Western blotting was performed to measure PSA levels. (**C**) FUS is recruited to the enhancer ARE III of the *PSA* gene. LNCaP cells were treated with 10 nM R1881 for 6 hrs. ChIP was subsequently applied and target DNA was amplified by qPCR. Values represent ratio of signals from anti-FUS ChIP to that of IgG isotype control. Columns = mean ± standard deviation. *P<0.05, n = 3.

To determine if FUS increases expression of endogenous genes, levels of protein and mRNA of known androgen-regulated genes were next measured. *PSA* mRNA and mRNA from five other androgen-inducible genes were significantly reduced upon depletion of FUS (Figure 5A). *TMPRSS2* mRNA was reduced by only one of the siRNAs, and the androgen-repressed gene, *CAMK2N1* was not affected by any of the siRNAs (data not shown). Consistent with reduced levels of mRNA, levels of PSA protein were significantly reduced in LNCaP cells upon depletion of FUS (Figure 5B). To determine if FUS was recruited to DNA with the AR in response to androgen, chromatin immunoprecipitation (ChIP) assay was performed. Immunoprecipitation of cross-linked protein-DNA complexes using an antibody to FUS followed by amplification of ARE III of the *PSA* gene revealed that FUS was recruited to this ARE (Figure 5C). Collectively, these data suggest that FUS enhances AR activity, at least at some target genes, consistent with the strong intrinsic transactivation function attributed to FUS (Fig. 3).

### Depletion of FUS reduces proliferation of LNCaP cells

**Figure 6:**
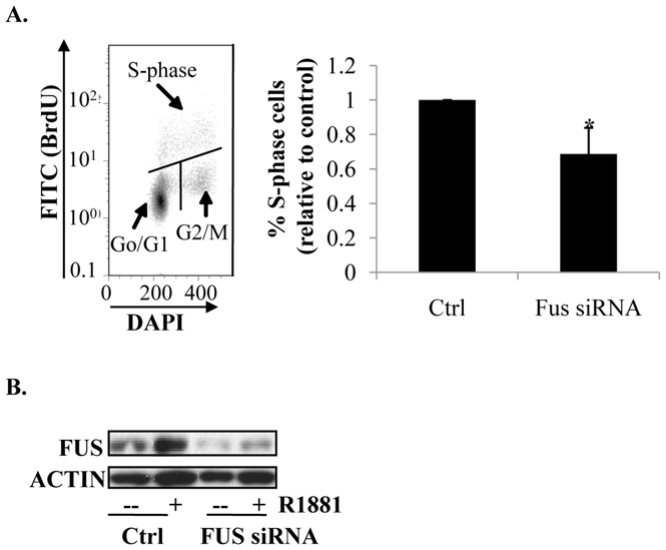
The effects of FUS depletion on proliferation of LNCaP cells. (**A**) Flow cytometric quantification of S-phase cells. siRNA-transfected LNCaP cells were treated with 0.1 nM R1881 for 48 hours. Cells were labelled with BrdU, stained with FITC-conjugated anti-BrdU antibody and DAPI, and subjected to flow cytometry. The graph on the right represents quantification S-phase cells as a ratio to the respective controls. *P<0.05, n = 3. (**B**) Levels of FUS protein in cells treated with FUS siRNA. Western blot analysis of levels of FUS and loading control actin corresponding to the samples whose cell-cycle profile is plotted in (A).

AR is essential for the growth of LNCaP cells [14]. To assess if decreased AR activity upon depletion of FUS would result in decreased proliferation, we performed cell-cycle analysis using flow cytometry. As expected, R1881 (0.1 nM) increased the population of cells in S-phase (data not shown) which was reduced by ∼30% upon depletion of FUS (Figure 6A). Noteworthy, the data was obtained from transient transfection of siRNA resulting in relatively inefficient depletion of FUS (Figure 6B). These data implicate a pro-growth function of FUS on androgen-dependent proliferation, which is consistent with increased AR transcriptional activity.

### Hormone-responsiveness of FUS expression

**Figure 7:**
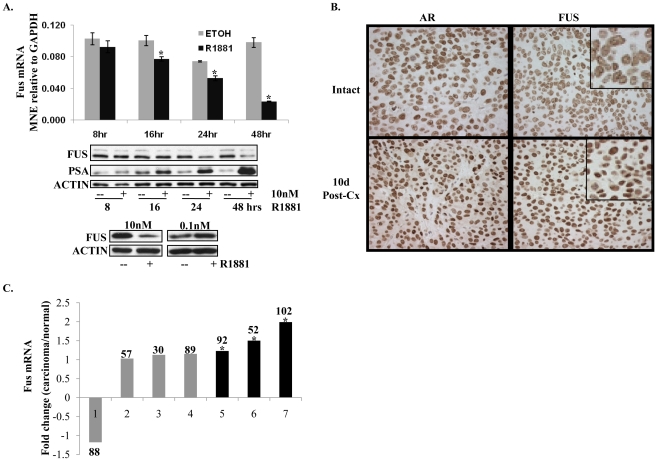
Regulation of FUS expression. (**A**) FUS mRNA levels in response to androgen treatment. Upper panel: FUS expression was measured by qRT-PCR in LNCaP cells treated with 10 nM R1881 for the indicated time points. Middle panel: western blot analysis of levels of FUS, PSA and Actin proteins at the indicated time points. Lower panel: western blot analysis of levels of FUS protein in LNCaP cells that were treated with 10 nM versus 0.1 nM for 48 hrs. Error bars = mean ± standard deviation. *P<0.05, n = 3. (**B**) FUS levels in response to hormonal status *in vivo*. LNCaP xenografts harvested from intact (non-castrated) and castrated mice (10 days post-castration) were stained for FUS and AR proteins. (**C**) FUS levels in clinical prostate cancer versus corresponding benign tissue. Levels of FUS mRNA in clinical samples of prostate carcinoma versus normal prostatic tissue were re-analysed from previously obtained data sets using Oncomine. Black bars indicate studies where FUS was significantly overexpressed in cancer versus benign tissue. *P<0.05.

Expression of some AR co-activators such as steroid receptor co-activators (SRCs) is modulated by androgens and/or AR [15]–[17] as part of what appears to be a network of feed-forward and feed-back mechanisms. FUS expression, at the mRNA (Figure 7A, upper panel) and protein levels (Figure 7A, middle panel), was repressed *in vitro* in LNCaP cells after 48 hrs treatment with 10 nM R1881. Treatment of LNCaP cells with 0.1 nM R1881 for equivalent period of time (48 hrs), however, had negligible effects on expression of FUS expression (Figure 7A, lower panel). Immunohistochemistry was employed to evaluate FUS gene expression in LNCaP xenografts. Tumor samples were prepared from intact (non-castrated) and castrated mice (10 days post-castration). Consistent with the *in vitro* data with 10 nM R1881, castration resulted in increased expression of FUS (Figure 7B).

### FUS expression tends to be higher in prostate carcinoma versus benign tissue

To determine if the expression of FUS is altered in cancer relative to benign prostate tissue, Oncomine database analysis was performed using seven different clinical cohorts. Expression of FUS was elevated in prostate carcinoma compared to benign tissue using data from three studies (Figure 7C). However, in four additional studies of comparable size, there was no significant difference between levels of FUS mRNA in prostate carcinoma compared to levels in benign prostatic tissue.

## Discussion

AR is essential for all phases of prostate cancer progression including the terminal CRPC stage. Underlying this role of AR is a dynamic interaction with a various co-activators and co-repressors. The scope of pathophysiologically relevant protein-protein interaction involving AR in prostate cancer cells is, however, not fully known. Here, we show for the first time that FUS interacted with AR endogenously in prostate cancer cells and further revealed the following: (1) FUS had intrinsic transactivation capacity in prostate cancer cells; (2) FUS enhanced AR-mediated transcription; (3) depletion of FUS reduced levels of expression of androgen-induced genes; (4) FUS was recruited to AREs in response to androgen; (5) reduction of levels of FUS limited androgen-induced proliferation; (6) levels of FUS were altered by androgen status *in vitro* and *in vivo*; and (7) levels of FUS were elevated in clinical samples of prostate cancer.

The DNA-binding domains (DBD) steroid receptors display high sequence homology (e.g. AR DBD has 77% and 57% sequence similarity with those of glucocorticoid receptor and estrogen receptor, respectively), and can interact with many of the same coactivators. Using recombinant proteins, FUS has been shown to interact with the DBDs of retinoid X, estrogen, thyroid, and glucocorticoid receptors, but AR was not examined [18]. Binding of FUS to DBDs of other hormone receptors did not interfere with DNA-binding activities, although the role of FUS in their transcriptional activities was not assessed [18].

Here, we demonstrate that FUS harbors strong transactivation domains that were functional in prostate cancer cells. The C-terminal region containing the RRM and RGG motifs of EWS can suppress the transactivation capacity of the NTD in rabbit kidney-derived RK-13 cells [19]. In contrast, here no suppressive function of the C-terminal region was observed when comparing full-length FUS to the FUS 1–273 fragment that lacks the C-terminal RRM and RGG motifs. Furthermore, FUS 274–526 that contains the RRM and RGG motifs but not the TAD, displayed a strong transactivation capacity. Similar structure-function relationship data involving FUS were previously reported using the human embryonic kidney 293 cells [20]. Thus, there are functional transactivation domains in FUS other than those found in its NTD at least in prostate cancer and 293 cells. Discrepancies in the literature between FUS and EWS transactivation domains may be cell-specific or reflect domain divergence (45% identity; 64% similarity) [5] between EWS (a 699 aa long protein) and FUS (a 526 aa long protein).

Expression of various co-regulators of AR is responsive to androgens perhaps as part of feed-back mechanisms [15]–[17]. Expression of FUS is responsive to hormone status in prostate cancer cells. At physiological levels of androgen, FUS expression was reported to be significantly reduced in LNCaP cells treated for 48–96 hrs with 10 nM mibolerone [21] as also shown here with 10 nM R1881 (Fig. 7A). However, a lower concentration of androgen, 0.1 nM R1881, did not significantly alter levels of FUS *in vitro*, and *in vivo* castrate levels of androgen were associated with increased levels of FUS. This expression profile resembles that of previously characterized AR co-activators that are modestly repressed by androgens [15]–[16]. Interestingly, repression of these co-activators by androgens is with a slower kinetics resembling that of androgen-responsive expression of FUS. Analysis of levels of FUS mRNA in various prostate and non-prostate cancer cell lines did not reveal systematic differences (Fig S3). Consistent with this, Oncomine data mining of microarrays from clinical samples of various solid tumor types did not show significant up or down-regulation FUS levels in prostate tumors relative to other tumor types (Fig S4). In contrast, comparison of mRNA data from benign versus adenocarcinoma prostate tissues revealed up regulation of FUS in three independent studies and no significant difference in four others. No study showed significant decrease of expression of FUS in prostate adenocarcinoma versus benign tissue.

AR transcriptional activity is required for the maintenance and growth of the prostate which forms the rationale for androgen ablation therapies for prostate cancer. Consistent with FUS being a coactivator of AR and its depletion reducing AR transcriptional activity and androgen-dependent growth as shown here, among the prominent phenotypes in FUS−/− mice is male sterility, and smaller male reproductive organs with apparent involution of some of these structures [22]. These phenotypes are reminiscent of AR−/− mice, or more refined domain- and cell type-specific *AR* gene disruptions [23]–[24]. Thus, it is possible that depletion of FUS decreases AR transcriptional activity in these classical androgen-dependent tissues in agreement with the data presented here. These data are in contrast to a previous report that FUS overexpression in LNCaP cells, using a tetracycline-inducible system and subsequent treatment with 10 nM mibolerone for four days, leads to emergence of sub-G1/apoptotic cells [21]. Intriguingly, no sub-G1/apoptotic cells were detected in the absence of androgen, with or without FUS overexpression [21]. Thus, in those studies FUS overexpression led to an androgen-inducible pro-apoptotic effect and changes in expression of cell cycle proteins [21].

Overall, while further studies are needed to resolve the role of FUS in prostate cancer disease progression, this study revealed that: **1**) FUS interacts with AR; and **2**) FUS enhances the transcriptional activity of AR.

## Materials and Methods

### Plasmids, cell culture and transfections

FUS expression plasmids [20], p5×Gal4UAS-TATA-luciferase [12], and AR expression vector, ARO, [25] have been previously described. PFR-LUC was obtained from Invitrogen, Carlsbad, CA, USA. AR reporter plasmid, PSA (6.1 kb)-luciferase contains the promoter/enhancer regions of the PSA gene and was provided by Dr. J.-T. Hsieh (University of Southwestern Medical Center, Dallas, TX). ARR3-tk-LUC contains three copies of androgen response region of the rat *probasin* gene fused to the basal thymidine kinase (TK) promoter [26].

LNCaP cells, obtained from Dr. Leland W.K. Chung (Samuel Oschin Comprehensive Cancer Institute, CA, USA), were grown in RMPI containing 5% Fetal Bovine Serum (FBS) and were used at passage 39–50. HEK293 cells were maintained in DMEM containing 10% FBS. Unless stated otherwise, cells were incubated in serum-free, phenol-red free media for 24–48 hrs prior to treatment with indicated concentrations of R1881. Transfection of LNCaP cells was performed using lipofectamine 2000 (Invitrogen) for experiments involving siRNAs or lipofectamine (Invitrogen) for all others as per manufacturer's instructions at 0.2–0.4% and 0.5% (v/v), respectively. FUS (HSC.RNAI.N001170634.11.7; HSC.RNAI.N001170634.11.8) and control siRNAs (Invitrogen) were used at 10–50 nM concentrations. HEK293 cells were transfected using lipofectamine 2000 at 0.4% v/v. Exact amounts of plasmids per transfection are indicated in Figure legends.

### Quantitative RT–PCR

Total RNA was prepared using the RNeasy mini kit (Invitrogen). Total RNA (0.5 µg) was reverse-transcribed via oligo-dT priming using the SuperScript III kit (Invitrogen). PCR was performed using gene-specific primers in the presence of SYBR Green Supermix (Invitrogen) using the ABI 7900 real-time PCR machine (Applied Biosystems, Foster City, CA, USA). The thermocycling protocol was as follows: at 95°C for 2 min, followed by 40 cycles of 95°C for 15 sec and 55°C for 30 sec. Ct (threshold cycle number) values were converted to mean expression values relative to *GAPDH* levels according to the Pfaffl method. FUS primers [5] and all other primers have previously been described [27]–[28].

### Immunofluorescence

Cells were washed with PBS and fixed in 1% paraformaldehyde in PBS for 30 min. Cells were permeabilized in 0.1% Trition X-100 in PBS twice for 5 min. After blocking for 30 minutes in 2% BSA in PBS containing 0.1% Trition X-100, samples were incubated with the indicated antibodies (1∶50) in blocking buffer for 1 hr. Cells were washed with blocking buffer 4 times and incubated with appropriate secondary FITC or rhodamine-conjugated IgG (1∶100) in blocking buffer for 45 min. After 4 washes with PBS containing 0.1% Trition X-100, DAPI-containing mounting solution was added. Slides were visualized using using Fluoview confocal microscope (Olympus, Markham, ON, Canada).

### Co-Immunoprecipitation and Chromatin immunoprecipitation assay

LNCaP cells (2–3×10^6^) were plated in 15 cm dishes with RPMI containing 5% FBS. After 24 hrs, media was changed into serum- and phenol red-free RPMI and the cells were incubated for additional 24 hrs. Cells were subsequently treated with R1881 (10 nM) or its vehicle (ethanol) for 6 hrs. Cells were rinsed with PBS before harvesting and then lysed with 1 ml of lysis buffer (20 mM Tris-HCl pH 7.8, 140 mM NaCl, 0.5% sodium deoxycholate, 0.5% Nonidet P-40) in the presence of a cocktail of protease inhibitors (Roche, Mississauga, ON, Canada). Lysates were passed through 27-G needles five times and were subsequently pre-cleared with protein A/G agarose beads (5% v/v) and a mixture of mouse and rabbit IgG (2 µg/ml each) for 1 hr. The resulting supernatant was incubated with the indicated specific antibodies including AR 441, AR C19, and FUS ( Santa Cruz Biotechnology Inc, Santa Cruz, CA, USA) at a concentration of 1–2 µg/ml for 16–24 hrs. Protein A/G agarose beads (5% v/v) were subsequently added and the mixture incubated for an additional 3 hrs. The beads were washed with 1 ml of the lysis buffer five times and then resuspended in 40–80 µl of standard SDS sample buffer.

Chromatin immunoprecipitation assay was performed as described [27]. Briefly, cells were treated with R1881 (10 nM) or vehicle for 6 hrs and were subsequently fixed with formaldehyde to cross-link chromatin. After sonication to shear chromatin/DNA, lysates were prepared and immunoprecipitated with the indicated antibodies. Target DNA was measured by real time PCR using the ABI 7900 real-time PCR machine (Applied Biosystems, Foster City, CA, USA). Values were normalized against signal from the respective IgG control.

### Western blot analyses

Samples were loaded into 10% Tris/glycine-based SDS-polyacrylamide gels. Proteins were transferred into polyvinylidene difluoride membranes (Millipore, Billerica, MA, USA) according to standard protocols using Tris/glycine-based transfer buffer in Bio-Rad's submarine system (Mississauga, ON, Canada). Blots were probed with following antibodies: AR PG21 (1∶1000) (Upstate Biotechnology, Waltham, MA, USA); AR 441 (1∶500), or FUS 4H1 (1∶1000) (Santa Cruz Biotechnology Inc).

### Proliferation assay

LNCaP cells (5×10^5^) in 10 cm plates were transfected with siRNAs using lipofectamine 2000 (Invitrogen). After 24 hrs, the media was changed into RPMI containing 10% charcoal-stripped FBS and cells were incubated for additional 24 hrs before treatment with 0.1 nM R1881 or equivalent amount of ethanol. Two days pos-treatment, cells were allowed to incorporate BrdU (Sigma, Oakville, ON, Canada) at 10 uM for 3–4 hrs. Subsequently, cells were harvested, washed with PBS and fixed with ethanol at −20°C for a minimum of 2 hrs. Cells were washed and their DNA was denatured with 2 N HCL, 0.5% Trition X-100 for 20 min. Following 4 washes, cells were blocked with 4% FBS/0.1% Trition X-100 in PBS and subsequently stained with 1∶40 ant-BrdU antibody. After two washes, DAPI was added at 2 ug/ml and samples were subjected to flow cytometry.

### In vivo experiments

Animal experiments were approved by the University of British Columbia Animal Care Committee (Permit No. A05-1794), and tissue processing thereafter was conducted as previously described [29]. Immunohistochemistry was performed using anti-FUS (1∶50) (4H1; Santa Cruz Biotechnology, Inc) and anti-AR (1∶50) (N-20; Cruz Biotechnology, Inc) and detection was based on alkaline phosphatase chemistry.

## Supporting Information

Figure S1 **Identification of FUS as AR -interacting protein by co-immunopreciptation followed by mass spectrometry (MS).** MS/MS spectrum of m/z 2251.86 (from 751.63, 3+) of FUS was unambiguously assigned the identified sequence APKPDGPGGGPGGSHMGGNYGDDR. Spectra for two other peptides are shown in Figure 1 and Figure S2.
(TIF)

Figure S2 **Identification of FUS as AR -interacting protein by co-immunopreciptation followed by mass spectrometry (MS).** MS/MS spectrum of m/z 1407.64 (from 704.83, 2+) of FUS was unambiguously assigned the identified sequence LKGEATVSFDDPPSAK. A third peptide spectrum is shown in Figure 1.
(TIF)

Figure S3 **Comparative analysis of FUS expression in various cancer cells.** Cell lines representing various cancer types were grown in their respective cell lines. Quantitative RT-PCR was performed to quantify FUS mRNA relative to GAPDH. LNCaP was used as reference for relative normalized expression. Prostate cancer cell lines that are AR positive are shown in red and in blue are AR negative prostate cancer cell lines. In black are non-prostatic cancer cell lines. Columns = mean ± standard deviation. *P<0.05, n = 3.
(TIF)

Figure S4 **Comparative analysis of FUS expression in clinical samples from various cancer types.** Data from a previous study is mined using Oncomine.
(TIF)
