# Supplementary figures and images for: FUS/TLS Is a Co-Activator of Androgen Receptor in Prostate Cancer Cells
Source: PLoS One. 2011 Sep 1;6(9):e24197. doi: 10.1371/journal.pone.0024197 (PMC3164714; doi:10.1371/journal.pone.0024197)

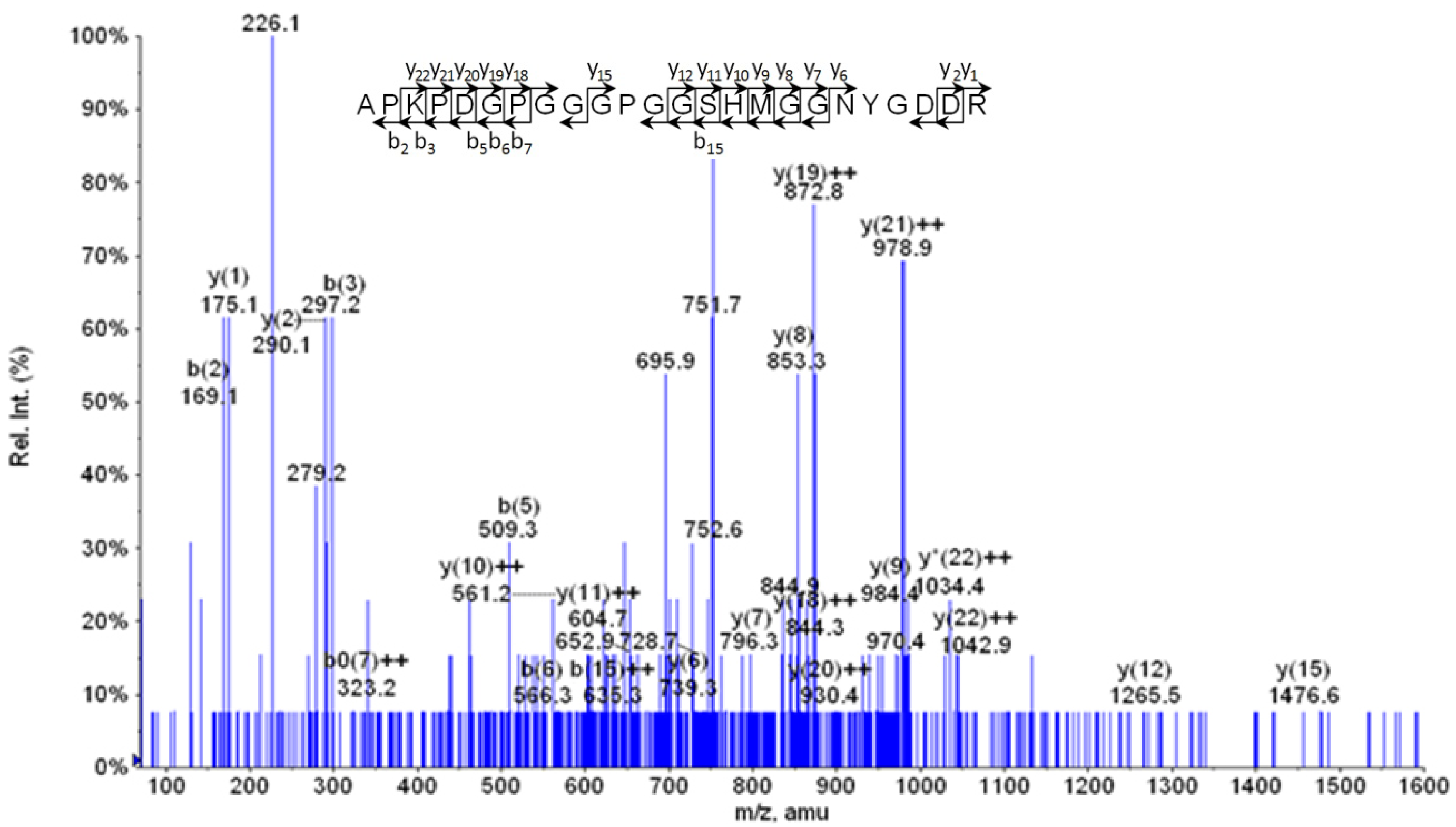

Supplement: Figure S1 — Identification of FUS as AR -interacting protein by co-immunopreciptation followed by mass spectrometry (MS). MS/MS spectrum of m/z 2251.86 (from 751.63, 3+) of FUS was unambiguously assigned the identified sequence APKPDGPGGGPGGSHMGGNYGDDR. Spectra for two other peptides are shown in Figure 1 and Figure S2. (TIF) [file pone.0024197.s001.tif]

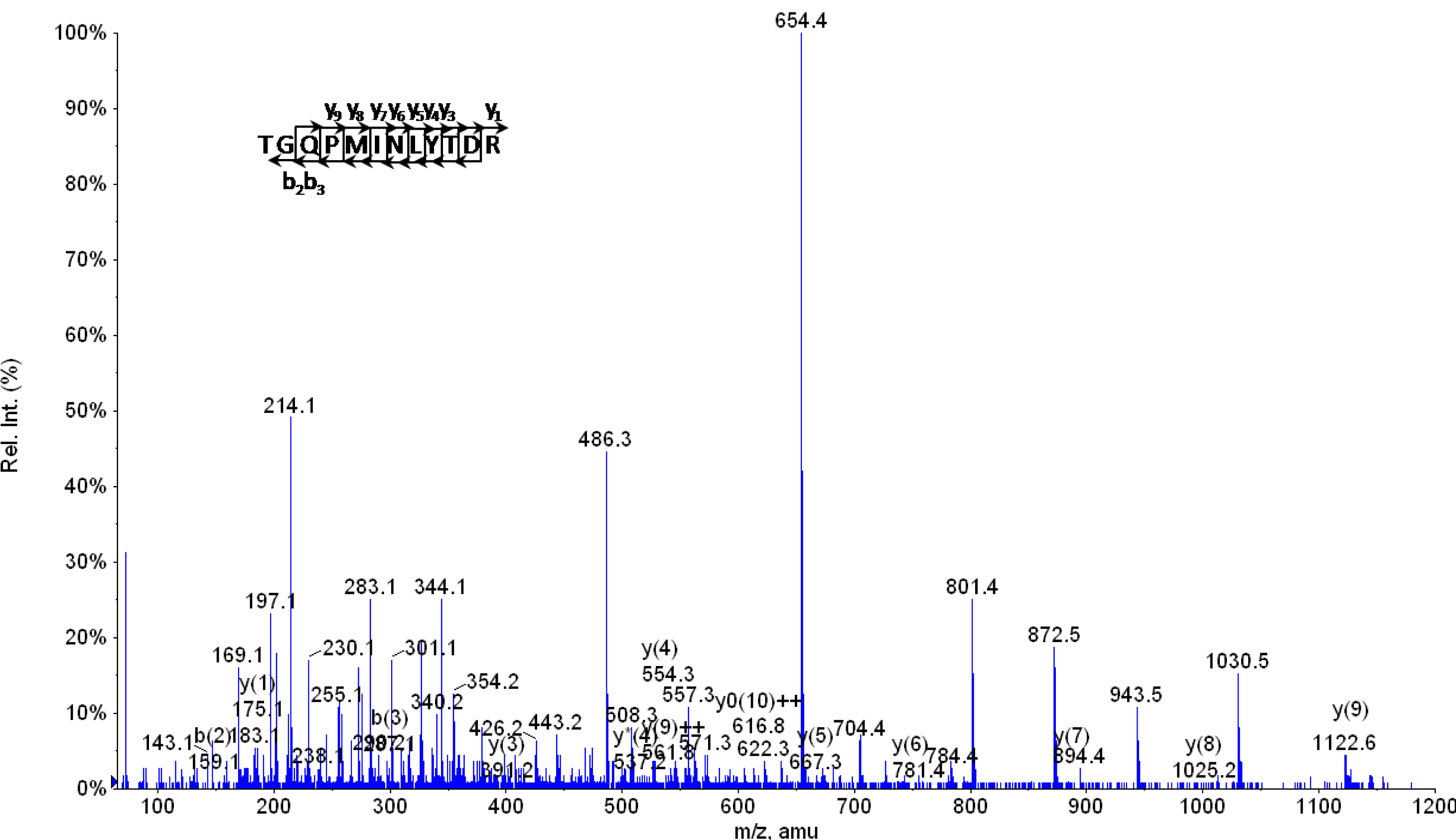

Supplement: Figure S2 — Identification of FUS as AR -interacting protein by co-immunopreciptation followed by mass spectrometry (MS). MS/MS spectrum of m/z 1407.64 (from 704.83, 2+) of FUS was unambiguously assigned the identified sequence LKGEATVSFDDPPSAK. A third peptide spectrum is shown in Figure 1. (TIF) [file pone.0024197.s002.tif]

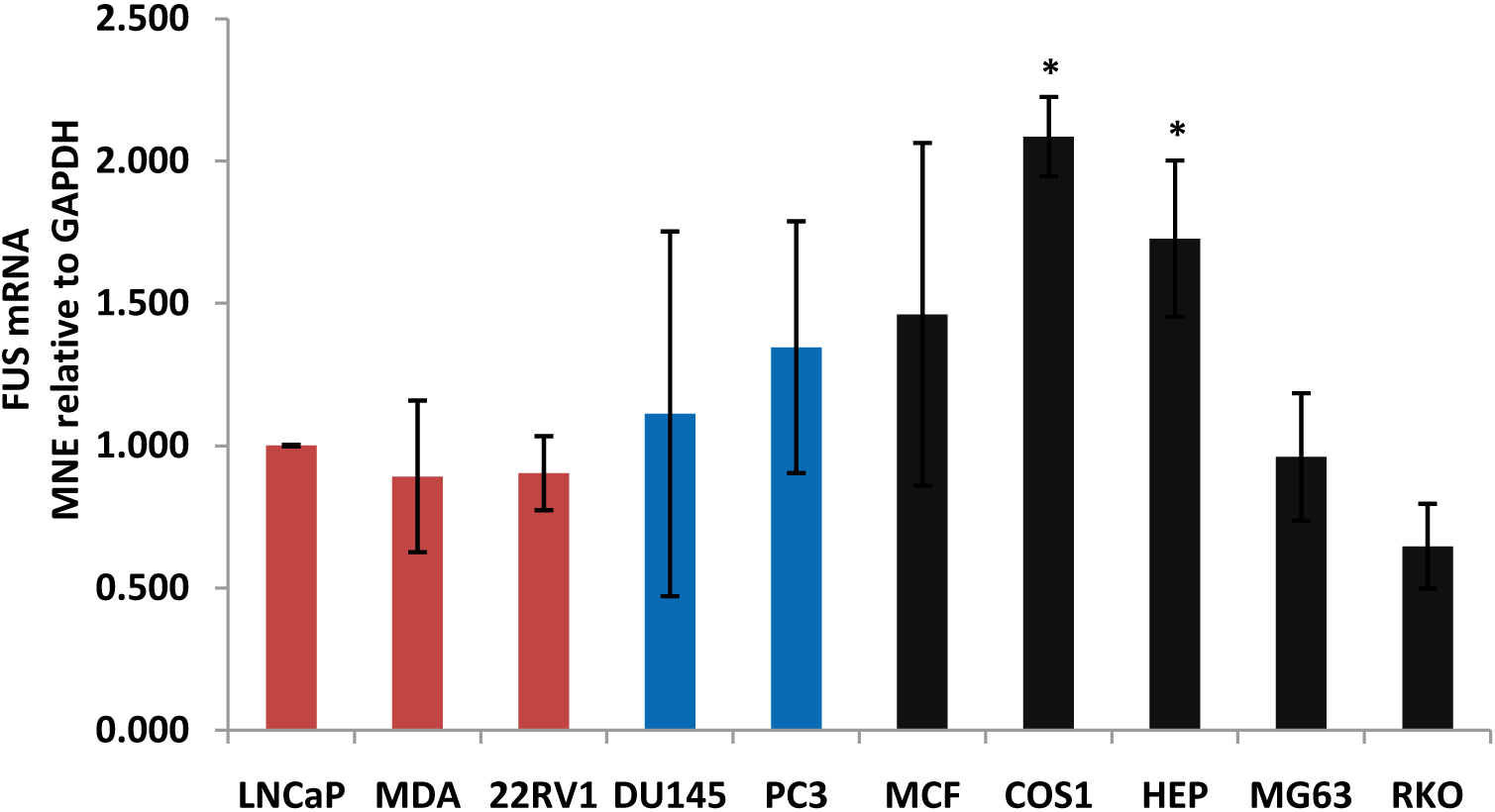

Supplement: Figure S3 — Comparative analysis of FUS expression in various cancer cells. Cell lines representing various cancer types were grown in their respective cell lines. Quantitative RT-PCR was performed to quantify FUS mRNA relative to GAPDH. LNCaP was used as reference for relative normalized expression. Prostate cancer cell lines that are AR positive are shown in red and in blue are AR negative prostate cancer cell lines. In black are non-prostatic cancer cell lines. Columns = mean ± standard deviation. *P<0.05, n = 3. (TIF) [file pone.0024197.s003.tif]

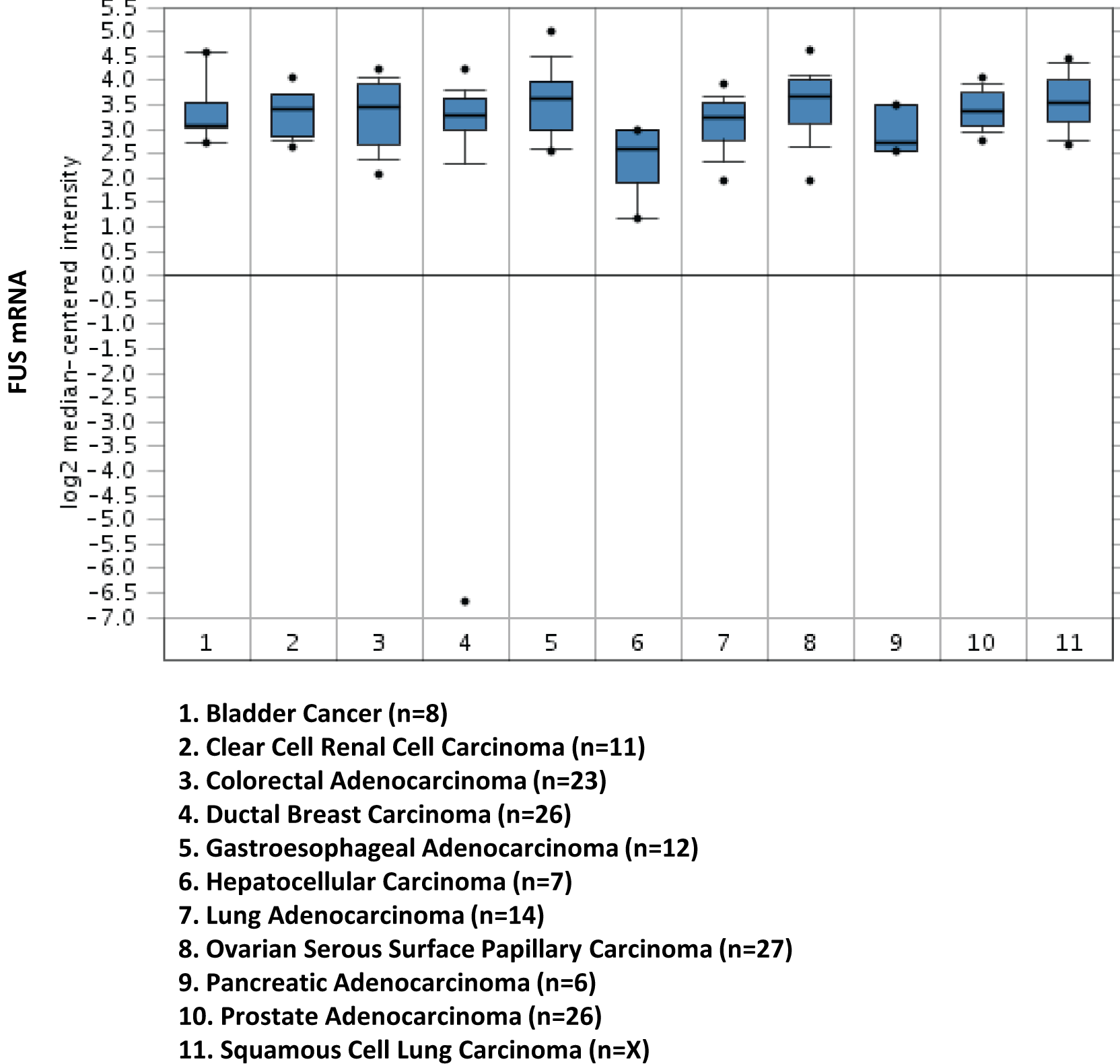

Supplement: Figure S4 — Comparative analysis of FUS expression in clinical samples from various cancer types. Data from a previous study is mined using Oncomine. (TIF) [file pone.0024197.s004.tif]
